# Neurodevelopment and Dietary Intake in Toddlers—A Cross-Sectional Study from the Healthy Children 2021 Project

**DOI:** 10.3390/nu15245105

**Published:** 2023-12-14

**Authors:** Micaela Cunha-Rodrigues, Rafaela Rosário, Ana Duarte, Maria José Silva, Cláudia Augusto, Mónica Rodrigues, Patrícia Padrão, Pedro Moreira

**Affiliations:** 1 Faculty of Nutrition and Food Sciences, University of Porto, 4150-180 Porto, Portugal; micaela.rodrigues97@hotmail.com (M.C.-R.); carolina101997@hotmail.com (M.R.); patriciapadrao@fcna.up.pt (P.P.); 2 School of Nursing, University of Minho, 4710-057 Braga, Portugal; anacspduarte@gmail.com (A.D.); mjsilva@ese.uminho.pt (M.J.S.); coliveira@ese.uminho.pt (C.A.); 3 Health Sciences Research Unit: Nursing (UICISA: E), Nursing School of Coimbra (ESEnfC), 3045-043 Coimbra, Portugal; 4 Nursing Research Centre, University of Minho, 4710-057 Braga, Portugal; 5 Research Centre on Child Studies (CIEC), Institute of Education, University do Minho, 4710-057 Braga, Portugal; 6 Epidemiology Research Unit and Laboratory for Integrative and Translational Research in Population Health, Institute of Public Health, University of Porto, 4050-600 Porto, Portugal

**Keywords:** neurodevelopment, bayley scales of infant and toddler development, dietary factors, food processing, unprocessed and minimally processed foods, dietary diversity

## Abstract

Little is known about the potential associations between neurodevelopment, dietary diversity and food processing in the toddler period. This study aimed to estimate the association between these dietary quality dimensions and neurodevelopment in toddlers. Data for this cross-sectional analysis came from the Healthy Children 2021 project and included 212 toddlers (51.9% females, aged 12–36 months) from 15 Portuguese childcare centers. Neurodevelopment was assessed through Bayley Scales of Infant and Toddler Development. Dietary intake was gathered by a two-day non-consecutive dietary recall. The food items were categorised with NOVA classification. Dietary diversity was explored through Minimum Dietary Diversity (MDD). Logistic regression models adjusted for potential confounders were performed. Girls with a higher energy contribution of unprocessed/minimally processed foods and with an above median MDD score had higher odds of achieving a higher neurodevelopment score (aOR:1.04; 95%CI 1.01; 1.08 and aOR:2.26; 95%CI 1.01; 5.06, respectively); no significant association was observed in boys. Our findings suggest that these dietary dimensions are associated with a higher neurodevelopment in toddler girls. This should be further studied as a possible early link between dietary factors and neurodevelopment. Promotion of healthy eating can be promising in improving neurocognitive development and might help to introduce public health recommendations for toddlers’ nutrition.

## 1. Introduction

The early childhood period is well recognised as a sensitive time window for the optimal growth, development and well-being of children [1]. During this phase, the relationships between cognitive development, motor development, language development, future educational and health outcomes are crucial [1,2] and are considered predictors of lifelong and academic achievement, wealth and quality of life [2]. On the other hand, early developmental deficits have been linked to an increased risk of chronic diseases in later life [3], unemployment and low socioeconomic positioning in adulthood [2].

Neurodevelopment is influenced by a number of factors ranging from gestational age at birth to biological, socio-economic and psychosocial environment. Nevertheless, there are important and controllable environmental factors that can profoundly influence early brain development [4]. In this current of thought, there is growing evidence that nutrition in the late foetal and early neonatal period has a significant impact on neurodevelopment [4]. Previous studies [4,5,6] have found that stunting or underweight, head circumference or body composition, undernutrition, specific vitamin A, zinc, iron and iodine deficiencies are associated with impaired developmental outcomes in young children. Others [7] have reported that healthier linear growth, as measured by a height-for-age Z-score, is linked with better cognitive, language and motor development. By contrast, complementary feeding routines are also a vital determinant of nutrition in infants and young children, while enhancing maternal bonding. This is particularly relevant considering that, in the last trimester of gestation and the first two years after birth, the brain is specially sensible to a poor and deficient diet [4,8], during which dietary patterns are also being developed and defined [9].

Over the last decades, children’s dietary patterns have suffered a dramatic change and have been characterised by a higher consumption of processed and ultra-processed foods with higher contents of saturated and trans fats, added sugars, food additives and salt. According to the latest data from the Portugal Childhood Obesity Surveillance Initiative (COSI) study [10,11] including children aged between 6 and 8 years old, 46.6% of the child population consumes between 1–3 times confectionery and pastry products per week, such as cakes, biscuits and sweets and 71.3% consumed sugary soft drinks up to three times a week [10]. Additionally, according to the National Food and Physical Activity Survey [12], 72% of children aged between 6 and 9 years old demonstrated inadequate consumption of fruit and vegetables. Further analysis reveals that 53% of the Portuguese population exceeds the recommended intake of saturated fats, with this percentage being notably higher in children (73%) [12] and the contribution of free sugars represents more than 10% of the total energy intake in 40.7% of Portuguese children [12]. Additionally, results from the pre-school children in four European cohorts, including a Portuguese birth-cohort (aged 6 months), showed that only 31.6% of Portuguese children aged three years and older eat five or more portions/day of fruit and vegetables, whereas the majority of them (67.4%) eat below five portions/day [12].

There is still lack of evidence about dietary pattern analyses that consider the nature, extent and purpose of food processing [13] and their diversity. Current understanding of the diet–cognitive development relationship in 12–36 months-old toddlers remains sparse and limited, since the majority of these studies have been focusing on older school children, where academic education could represent an important confounder. Therefore, optimizing dietary patterns during this period represents a golden opportunity to impact neurodevelopment across an individual’s lifespan [4] and also to advocate feasible targets for early prevention, education and intervention by parents, caregivers and childcare teachers.

Taking this into consideration, this study aimed to estimate the association between two dietary quality dimensions—energetic contribution to total energy intake from unprocessed and minimally processed foods and dietary diversity—and neurodevelopment in toddlers.

## 2. Materials and Methods

### 2.1. Study Participants and Design

**Figure 1:**
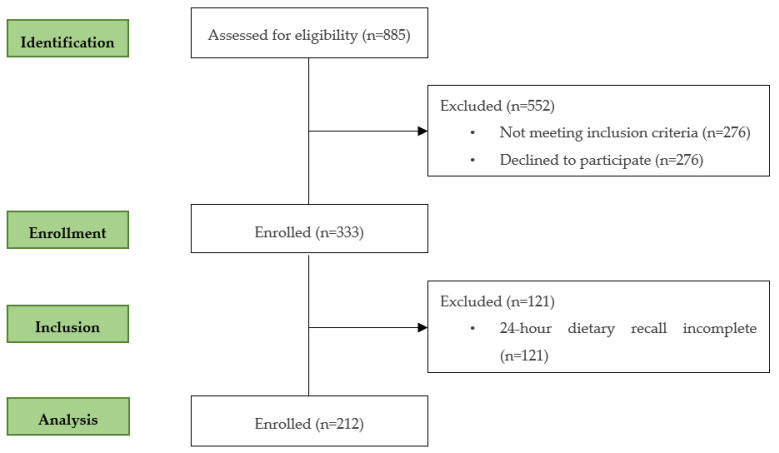
Flow of participants through each stage of the program.

Data for this cross-sectional study came from the Healthy Children 2021 project—a cluster randomized control trial conducted in the north of Portugal. During 2019, 15 out of 22 childcare centers from Braga, with a minimum of 20 children aged between 12 and 36 months were initially approached by phone and by email, were provided with a summary of the study and were invited to participate. The inclusion criteria were children aged between 12–36 months, and the exclusion criteria included children who received any local or systemic treatment likely to affect the evaluation of the study parameters, for example, children with chronic problems expected to influence length/height, weight or physical activity and children with diagnosed learning difficulties. Baseline measures were undertaken in Autumn 2019. Prior to data collection, informed consent was obtained from parents or caregivers according to the ethical standards laid down in the Declaration of Helsinki. Ethical approval was obtained from the Ethics Subcommittee of Life and Health Sciences Research (SECVS) (University of Minho) and registered at clinicaltrials.gov (NCT04082247). The flow of the subjects during the study is presented in Figure 1. Of the 885 children assessed for eligibility, 552 were excluded (276 because the parents refused to participate and 276 for not meeting the inclusion criteria), and 333 children were enrolled in the cross-sectional analysis.

Of the 333 children, 121 were excluded for not having the 24 h dietary recall completed. At the end, 212 toddlers were analysed in this cross-sectional study.

### 2.2. Bayley Scales of Infant and Toddler Development

Cognitive development was assessed using the Bayley Scales of Infant and Toddler Development—Third edition (Bayley-III) [14]. This is a comprehensive tool used to measure the developmental functioning of infants and young children between the ages of 12 to 42 months and to identify development issues during early childhood. The present scale involves five major developmental domains. Only the Bayley cognitive domain was used in this cross-sectional study and was conducted through direct observation of the child in test conditions. This subscale involves different dimensions, namely attention and habituation tasks (e.g., habituates to object); simple problem solving (e.g., object relatedness); object assembly; play and memory tasks; concept formation and grouping (e.g., concept grouping: colour). Finally, the Bayley cognitive subscale was applied and scored following the procedures described in the manual, according to the child’s age at the starting point [14].

In relation to general testing guidelines for the cognitive subscale and regardless of location, the testing environment was free of distractions with a quiet and comfortable room. Therefore, during the test application, lighting that might shine directly into the toddler’s eyes or loud noises that might distract them were avoided. A friendly environment was created by using a serene conversational tone of voice, encouraging interest in the tasks and reinforcing toddler’s efforts. 

Finally, and regarding the types of quantitative scores available, Bayley-III comprises the Raw Scores. However, comparisons between a child’s score and others are truthfully based on derived scores, which includes aged-based scaled scores, the one used in this analysis. They are derived from the total raw score and are scaled within a range of 1 to 19, a mean of 10 and a standard deviation (SD) of 3. 

### 2.3. Dietary Intake

Dietary intake was gathered by a two-day dietary recall from non-consecutive days, obtained and completed by the parents or/and educators. During its application, all food and beverages were described in detail, as well as commercial brands and culinary methods. Information regarding the place and time of consumption was also the subject of a question. Portion sizes were estimated using a book [15] and household measures (cups, glasses, food wrappers or containers) as an aid. Subsequently, energy and nutritional intake were estimated using the nutritional analysis software Food Processer Plus (version 11.9, ESHA Research Inc., Salem, OR, USA), which encompasses databases of Portuguese nutritional food compositions. During the introduction of 24 h dietary recalls on Food Processor Plus, the researcher also added recipes retrieved from all the recruited childcare centers.

The data collected from the two-day 24 h dietary recalls were analysed as mean. Breastfeeding was categorised as a dichotomic variable, and it was considered that toddlers were breastfed when “breastfed” appeared in the two-day 24 h dietary recalls. In relation to infant formula, a dichotomic variable was created and considered that toddlers consumed infant formula when “infant formula” was in the 24 h dietary recalls. For this variable, the researcher also added the nutritional profile of all the specific infant formulas mentioned in the 24 h dietary recalls (e.g., Nan, Aptamil, Nutribén…) and formed a continuous variable used as a percentage of the energetic contribution (%kcal) of infant formula to total energy intake.

### 2.4. Food Processing

The food and beverage items were categorised using the NOVA classification system [16], which groups them according to the extent and purpose of processing they undergo into four groups: unprocessed and minimally processed foods (NOVA 1), processed culinary ingredients (NOVA 2), processed foods (NOVA 3) and ultra-processed foods (NOVA 4). The first NOVA group refers to the edible parts of plants (fruits, leaves, seeds, roots, tubers) and animals (muscle, fat, organs, viscera, eggs, milk); fungi, algae and water. These foods are obtained directly from the environment and do not undergo any alteration or process, unless you count their removal from nature. Minimally processed foods are natural foods that undergo methods that have as their main purpose the preservation of the food (e.g., drying, crushing, grinding, filtering, roasting, boiling, non-alcoholic fermentation, pasteurization, chilling, freezing). In these cases, no oils, sugars or salt are added to the original food. The processes previously mentioned are designed to preserve natural foods, prepare them for storage and make them edible and safe for human consumption. The second NOVA group refers to products derived directly from nature or from group 1 foods by pressing, refining, grinding, milling and spray drying. They are typically used to prepare, cook or season unprocessed or minimally processed foods. The third NOVA group includes products manufactured by the food industry that are usually made by adding ingredients from the second group to foods from the first group to increase its durability or palatability. Lastly, the fourth NOVA group refers to industrial formulations mostly made from substances extracted from foods (fats, oils, sugar) or synthetized in laboratories from food extracts or other organic sources.

For each toddler, all food described in the two-day 24 h dietary recalls was allocated into the four major NOVA groups previously described. The quotient between the sum of each NOVA group and the estimated value of the total energy intake was calculated. Energy contribution (%) for each NOVA group to total energy intake was obtained as mean.

Since NOVA classifies infant formulas as ultra-processed foods, we decided to do the procedure formerly described without the energy contribution of infant formulas to total energy intake as well, because it could provide a protective effect derived from NOVA 4.

### 2.5. Dietary Diversity

Dietary diversity was assessed through the Minimum Dietary Diversity (MDD) [17]. This is a population-level indicator designed by the World Health Organization for infants and young children aged 6–23 months to assess their dietary diversity. The WHO 2010 document describes seven groups: grains, roots and tubers; legumes and nuts; dairy products; flesh foods; eggs; vitamin A-rich fruits and vegetables and other fruits and vegetables. The consumption of any quantity of food from each food group is sufficient to count, unless an item is only used as a condiment. This methodology allowed the creation of a dichotomous indicator based on whether or not the toddlers have consumed at least four out of seven defined groups. It was also taken into consideration the number of food groups the toddler consumed. Although the age range for this indicator is 6–23 months, the Global Nutrition Monitoring Framework Operational Guidance for Tracking Process in Meeting Targets for 2025 affirms that the age range could be further expanded [18].

### 2.6. Sociodemographic Data

Social, demographic and family profiles were assessed at baseline using the Graffar Scale [19], adapted to Portugal. The Graffar Scale is an international social classification that includes five criteria: occupation, level of education, sources of family income, housing comfort and appearance of the neighbourhood. From this questionnaire we retrieved the mother’s level of education.

### 2.7. Sleep Patterns

Parents were asked to report on their child’s total sleeping time per day and night. Sleep quality was assessed with Tayside Children’s Sleep Questionnaire [20], which evaluated the child’s ability to initiate and maintain sleep. This 10-item scale includes nine questions designed to be summed. The total score ranges from 0 to 36 with higher scores indicating greater severity of sleep problem. The tenth question aims to assess parents’ perception of the issue, but the data related to this question were omitted from the analysis.

### 2.8. Anthropometry

Toddler’s length/height and weight were measured by trained researchers with standardised procedures [21]. Measurements were taken with no shoes and wearing light clothing. For children aged 12–24 months, we measured their recumbent length while they were lying down. We used an infant stadiometer positioned on a stable, flat surface for this purpose. In cases where a child could stand but refused to lie down, we measured their standing height and then added 0.7 cm to obtain an equivalent length measurement [22]. For those toddlers older than 24 months, height was measured when they were standing straight against a portable stadiometer (Seca 254, Hamburg, Germany) and positioned in the Frankfort’s position. The values were pointed out to the nearest 0.1 cm. Body weight was measured through a portable electronic scale (Seca 254, Hamburg, Germany) to the nearest 0.1 kg. 

### 2.9. Statistical Analysis

The characteristics of the participants are presented for the whole sample by sex and by Bayley’s cognitive categories as percentages for categorical, as mean ± standard deviation for normal distributed continuous variables and as median (25th–75th percentile) for non-normal distributed continuous variables. Skewness and kurtosis test were used to check normality for continuous variables. Differences between groups were assessed through student’s *t*-test for independent and continuous normally distributed variables, whereas the Mann–Whitney test was performed for non-normally distributed. The Chi-squared test was used for categorical variables. 

Cognitive development and the energetic contribution to total energy intake of each NOVA group were categorised using the median cognitive scaled score of Bayley Scales—below median score (<10) and above median score (≥10)—and the mean energetic contribution for each NOVA category, respectively.

Dietary diversity was categorised using the median score of Minimum Dietary Diversity—below median score (<5) and above median score (≥5). Logistic regression models were applied to assess the association between NOVA 1 and dietary diversity (independent variables) with cognitive development (dependent variable), further adjusting for potential confounders based on the literature evidence (age, BMI, breastfeeding, infant formula, toddler’s sleeping time and mother’s education). Due to the perceived fragility in constructing the “breastfeeding” variable, models without adjustment for this variable were also conducted. Additionally, models including and excluding the percentual energetic contribution of infant formulas, for the reasons previously mentioned in the methods section, were also constructed.

Collinearity statistics were used to examine collinearity among all variables. A 0.05 level of significance and 95%CI were used. The analyses were performed through the IBM SPSS statistical package software v27.0 (IBM, Armonk, NY, USA).

## 3. Results

The main characteristics of participants by sex are presented in Table 1. The mean age of toddlers was 23.77 (±6.33) months and 51.9% (*n* = 110) were girls. The mean cognitive scaled score was 10.04 ± 2.72 and 9.56 ± 2.60 for girls and boys, respectively. In relation to Bayley’s cognitive categories, 59.1% of girls and 53.9% of boys were above the median cognitive scaled score with no significant differences found among boys and girls.

Table 2 presents the summary of dietary information by sex. NOVA 1 was the group that most contributed to total energy intake (57% and 60%), followed by NOVA 4 (23% and 19%) and NOVA 3 (12% and 13%) when including and excluding %kcal of infant formulas, respectively. No significant differences were found among girls and boys for the contribution of each NOVA group, except for NOVA 4, where boys had a significantly higher energetic contribution to total energy intake of ultra-processed foods than girls (25.29 ± 14.73 and 20.44 ± 13.93, *p* = 0.016), even when %kcal of infant formulas were excluded from the total energy intake (21.15 ± 12.44 and 16.55 ± 12.05, *p* = 0.007, respectively). A total of 59% of toddlers had a minimum dietary diversity score above median, with no significant differences between girls and boys.

The association between energetic contribution to total energy intake of NOVA 1 and the toddlers’ neurodevelopment is presented in Table 3. Girls with a higher energy contribution of unprocessed and minimally processed foods had higher odds of achieving a higher cognitive development score (aOR: 1.04; 95%CI 1.01; 1.08), when %kcal of infant formulas were excluded (model 1, crude). However, the same association was not observed in boys.

These results remained for the other model conducted where the percentage of energetic contribution (%kcal) of infant formulas were included: aOR: 1.04; 95%CI 1.01; 1.08 (model 2, crude). After adjustment for sex, age, breastfeeding, percentual energetic contribution to total energy intake of infant formulas, toddler’s sleeping time and mothers’ education, the same association was observed only for girls (aOR: 1.06; 95%CI 1.01; 1.11, models 1 and 2, adjusted); (aOR: 1.05; 95%CI 1.00; 1.11, models 3 and 4, adjusted).

The association between minimum dietary diversity and toddlers’ neurodevelopment is presented in Table 4. Girls who scored above the median minimum dietary diversity had higher odds of achieving a higher cognitive development score (aOR: 2.26; 95%CI 1.01; 5.06) (model 1, crude). However, the same association was not observed for boys.

The results remained after adjustment for all the confounders previously mentioned (aOR: 3.15; 95%CI 1.05; 9.48) (model 1, adjusted). Finally, when the model was not adjusted for breastfeeding (model 2, adjusted), the association remained only for girls too (aOR: 3.18; 95%CI 1.12; 9.08).

## 4. Discussion

This study found a positive association between neurodevelopment score and energetic contribution to total energy intake from unprocessed and minimally processed foods. Moreover, it revealed a positive association between neurodevelopment score and dietary diversity in girls. To our knowledge, there are few, if any, reports on whether and how these two dietary factors are associated with neurodevelopment in 12–36-month-old toddlers. In line with our findings, and despite the methodological differences on outcome assessment, other studies have proposed the beneficial effect of a diet enriched with healthy foods on neurodevelopment. A systematic review [3] conducted to evaluate whether healthier dietary consumption among children and adolescents impacts executive functioning verified that there was a positive association between healthier foods (e.g., fruits and vegetables, whole grains, fish) and executive functioning. Within the review mentioned before and more specifically Riggs et al. [23], both found that fruit and vegetable intakes were positively associated with executive functioning. Haapala et al. [24] observed a positive relation among fruit consumption and higher cognitive performance. Other findings demonstrated strong associations between some dietary factors, such as the intake of fish, fruits and vegetables and neurodevelopment [25] in the same-range toddler period as our study. Gale et al. [26] observed that children who had a dietary pattern characterised by higher intakes of fruit, vegetables and home-prepared foods in infancy increased slightly, after four years, in test scores of full-scale and verbal intelligence. Although none of the studies previously mentioned refer specifically to “unprocessed and minimally processed foods”, it is possible that those food groups are categorised as such [16,27], since all of them are edible parts of plants or animals or passed through processes that are intended to preserve them, make them safe or edible. Therefore, in an adequate variety, balance and combination, unprocessed and minimally processed foods—NOVA 1 group—are the basis for healthy diet patterns and food products [16] and are considered key dimensions of diet quality. In this current of thought, addressing specifically dietary patterns and not only food groups, the Mediterranean diet is a cultural model originating from the interplay between natural food resources and healthy eating practices [28]. This dietary pattern highlights the preference for local, seasonal, fresh and minimally processed foods [29]—NOVA 1 group—and emphasizes the low environmental impact of food production, processing and long-distance import food transportation [29]. In relation to adherence to the Mediterranean dietary pattern and neurodevelopment, Cornejo-Esteban et al. [30] found that the group with increased adherence had significantly higher scores in all of the academic indicators compared with the decreased adherence group in 6–18-year-old children and adolescents. Granziera et al. [31] observed that five-year-old children with maximum adherence to this dietary pattern had higher performance scores than those with low and moderate adherence. The authors also found positive effects for high intakes of vegetables on personal–social scores and a high unsaturated/saturated ratio on hand-eye coordination scores. These results are in line with ours since we observed a positive association between a high energetic contribution to TEI from NOVA 1 and toddler’s neurodevelopment, more specifically in the cognitive subscale, where girls with higher %kcal of NOVA 1 had higher odds of achieving a higher neurodevelopment score. However, the same association was not observed in boys.

Breastfeeding is unquestionably the best feeding method and the most complete source of nutrients for babies during their first six months, and the WHO [32] recommends exclusively breastfeeding for this period and continuously for up to two years in a complementary way. The role of breastfeeding in neurodevelopment is well established. Human breast milk provides all the nutrients required for brain development [33,34], and the physical and socioemotional contact between the mother and the child during breastfeeding can also stimulate neurodevelopment [35]. Therefore, breastfeeding is considered a relevant confounder in our outcome assessment. In our study, breastfeeding was only assessed through two-day 24 h dietary recalls, leading to the creation of a dichotomic variable. It was considered that toddlers were breastfed when “breastfed” appeared in those food records. Because 24 h dietary recall only refers to food and beverage items consumed in the prior 24 h, this may mean that toddlers can be categorised as not being breastfed when in reality they were. However, we took some precautions by conducting models without the adjustment for breastfeeding. Consequently, the results showed the same positive association between NOVA 1 and neurodevelopment in girls (aOR: 1.05; 95%CI 1.00; 1.11). Nevertheless, and although breastfeeding is highly recommended, not all mothers are able or willing to do so, and it may not be possible, suitable or solely adequate. In such cases, infant formulas (IF) are the only appropriate substitute for human milk [34]. In fact, IFs have been developed to mimic breast milk’s nutritional composition as closely as possible. NOVA classifies infant formulas as ultra-processed foods [27]. In reality, they are processed foods with a characteristic formulation of milk proteins, lactose or other sugars, vegetable oils, micronutrients and some other additives [36]. However, it is established that when breastfeeding is not possible, this substitute form of nutrition is required. Taking into account what was previously described, we have the necessity to study the association between NOVA 1 and neurodevelopment, without the percentual energetic contribution to TEI of infant formulas, which were within the ultra-processed foods (NOVA 4). To ensure that NOVA 1 would not be influenced by NOVA 4 because of the probable protective effect of %kcal from infant formulas, we conducted models including and excluding the energy contribution to TEI from this food product. Our results showed that the positive association between NOVA 1 and neurodevelopment was persistent (aOR: 1.04; 95%CI 1.01; 1.08, including %kcal of infant formulas; aOR: 1.04; 95%CI 1.01;1.08, excluding %kcal of infant formulas) (Table 3). This last model was also adjusted for the percentage energetic contribution to total energy intake of infant formulas.

As described before, unprocessed and minimally processed foods vary in energy density and in their content of macro and micronutrients such as vitamins, minerals and other bioactive compounds and no single type of food can provide humans with all necessary energy and essential nutrients in adequate balance [16] (except human breastmilk in the first six months of life). Having a high energetic contribution to total energy intake from NOVA 1 does not mean that toddlers have at the same time a high dietary diversity. This dietary dimension is also a well-recognised indicator reflecting the quality of complementary foods.

Regarding this other dietary dimension, our study found that girls who scored above the median minimum dietary diversity score (≥5 groups) had higher odds of achieving a higher neurodevelopment score (aOR: 2.26; 95%CI 1.01; 5.06). These results remained even after adjustment for confounders (aOR: 3.15; 95%CI 1.05; 9.48). In line with our findings, Zhao et al. [37] observed that 6–23-month-old children consuming at least five food groups had a 39% lower risk of poor development, more precisely the gross motor, fine motor, problem-solving and personal social subscales, compared to those consuming fewer than five food groups. One longitudinal cohort study [38] showed that each day consuming the minimum dietary diversity groups counted on the way to a 35% reduced risk of being in the lowest category of developmental scores.

The relationship between these two dietary factors and toddler’s neurodevelopment outcomes might be clarified by several mechanisms. Diverse studies [3,4,8,39,40] have reported that early brain development, both structural and functional, is highly dependent on an optimal supply source of dietary macro- and micronutrients that are essential for optimal growth, immune response and cognitive function, namely protein, iron, zinc, iodine and vitamin B12. Iron and zinc are crucial micronutrients for adequate development of the hippocampus and prefrontal cortex in the first 1000 days of life, and vitamin B12 is a key factor for normal brain and nervous system function [41]. All of these can be obtained through a diet based on unprocessed and minimally processed foods, rich in those nutrients and diversified to capture all the sources of those nutrients. Dietary diversity is an indicator of micronutrient deficiencies and toddlers consuming a higher diversity of foods are more likely to be protected from those micronutrient deficiencies [37]. Another biological explanation is that complex phenols with critical antioxidant properties, such as fruit, vegetables, berries, olive oil, vitamin C and E and carotenoids are found in high concentrations in diets with these two quality dimensions and they have been shown to protect the brain against neuronal damage by decreasing inflammation and oxidative stress and by supporting cell proliferation [24,42]. Additionally, flavonoids are the principal element in diets with these characteristics and most of their own actions are related to their antioxidant proprieties, including suppression of reactive oxygen species (ROS) formation, scavenging those reactive oxygen species and upregulation of antioxidant defences [24,42]. In summary, some lines of evidence suggest that healthy subjects consuming a diet containing these dimensions have higher plasma levels of lipophilic antioxidant micronutrients, lower levels of biomarkers related to oxidative stress and better scores on neurophysiological evaluation [42]. Moreover, low intake of saturated fats and high intake of flavonoids may increase circulating concentrations of brain-derived neurotrophic factor [42,43], which is a growth factor that has been shown to enhance synaptic plasticity, neurogenesis, neural survival, learning and memory. Finally, the intake of docosahexaenoic acid, which is found in fish, has been directly associated with endothelial nitric oxide synthesis and may therefore expand blood vessels and increase cerebral blood flow [42]. Another possible explanation might be that the dietary pattern of toddlers with high consumption of unprocessed and minimally processed foods and high dietary diversity has a positive effect on brain development during this special time when brain growth velocity is high [26].

Exploring another dimension, an alternative explanation for these results is that toddlers who consume a higher diversity of foods have the opportunity to explore and interact with a wider variety of foods through their numerous textures, forms, sizes, shapes, colours, flavours and smells, allowing them to be exposed to a greater amount and variety of psychosocial stimulation [37,44]. While at home and at the childcare centers, dietary diversity may increase the amount of stimulation through some mechanisms: first, it is well known that the child’s and mother’s diet are correlated [45], and the latter is often the primary source of stimulation; and second, a mother who supplies a diverse diet to her child is also expected to supply diverse stimulation, having not only the time for feeding them but also playing and bonding with them.

Entering this circle, toddlers who often rely on the primary caregiver to prepare their food, feed them and interact with them are reinsured with a confident and enriched environment which promotes a vital exploration and interaction with peers, time and space in a secure way, thereby increasing toddler’s motor abilities, their physical activity, initiative, and leadership, contributing to the development of their problem-solving skills. The resulting motor skills allow toddlers to provide their own stimulation, leading to a more active child who is more likely to receive attention, be spoken to, and be heard by others.

All this, together, allows an enhancing process of their mental development, particularly when their experiences are mentally challenging, which is consistent with other literature [46] showing that a responsive and stimulating environment creates appealing situations for toddlers to develop their cognitive capacity.

In our study, only girls demonstrated a positive association between high energetic contribution to total energy intake of unprocessed and minimally processed foods and high dietary diversity and neurodevelopment. In line with our findings, other studies have found the same sex differences [24,26,47,48]. We observed that boys had a higher energetic contribution to total energy intake from ultra-processed foods than girls (25.29 ± 14.73 and 20.44 ± 13.93, *p* = 0.016), even when infant formulas were excluded from the total energy intake (21.15 ± 12.44 and 16.55 ± 12.05, *p* = 0.007, respectively). There is some evidence that male brains are more susceptible to stress than female brains [49]. Having a high energetic contribution to total energy intake from ultra-processed foods could be a chemistry stress source and thereby a plausible explanation for not finding a positive association between NOVA 1 and dietary diversity on neurodevelopment in boys. Furthermore, cerebral and grey matter volume in the frontal and parietal cortices of the brain reach their peak earlier in girls than in boys, as shown in a literature revision by Patton and Viner, regarding puberty [50] and in a cross-sectional study by Koolschijn et al. in individuals from 8 to 30 years old [51]. Although the reported results regard older individuals than those of our sample, it is possible that differences in brain maturation could be observed in toddlers, and this may possibly contribute to explain the stronger association of dietary factors with cognition in girls than in boys in the present study sample. Even so, possible sex differences merit consideration in future studies and are highly warranted. Finally, a literature review suggests that boys’ cognitive development benefits more from dietary interventions during infancy than girls [26].

Although it is not the major aim of our study, we could not fail to comment that NOVA 1 was the group that most contributed to total energy intake (57% and 60%), followed by NOVA 4 (23% and 19%) and NOVA 3 (12% and 13%) when including and excluding %kcal of infant formulas, respectively. This means that ultra-processed foods were the second source of energetic contribution to the daily energy intake of toddlers, even when %kcal of infant formulas was excluded. In line with these findings, the UPPER study [13] found that about half of Portuguese children and adolescents were classified as having an “unhealthy” dietary pattern, which was characterised by a higher intake of ultra-processed foods and free sugars, concluding that one-third of the energy consumed by Portuguese children and adolescents came from this food group. These results are particularly concerning since Portuguese dietary guidelines recommend not offering processed and ultra-processed foods during the first year of life, and additives like salt and sugar are expressly contraindicated.

One limitation of our study is the cross-sectional design that does not allow for establishing causal relationships. Therefore, the results from our study cannot illuminate the mechanisms that link these dietary factors and toddler’s neurodevelopment. Secondly, we take into consideration some confounders found in the literature, such as sex, age at cognitive assessment, parental level of education, breastfeeding, consumption of infant formula and toddler’s sleeping time. However, we cannot exclude the potential impact from other unmeasured and relevant covariates, such as smoking and drinking habits during pregnancy, drugs and nutritional supplements used during pregnancy, child’s birth weight, preterm or term birth, gestational age at birth, early infections and relevant medical story. Additionally, we did not have or include adequate proxies for some crucial factors related to neurodevelopment, like mother–child interaction and stimulation, the nature of the home environment or general social support. Also, as already mentioned above, we emphasize the limitations of the construction of “breastfeeding” and “infant formula” variables. Moreover, toddlers from families with low socio-economic status are more likely to consume a low-quality diet, and it is possible that this variable may act as an achievement buffer, thereby partially explaining the relationship between diet and neurodevelopment. Hence, we adjusted for the mother’s education in the present study. Still, it is important to account that the socioeconomic status, measured comprehensively (i.e., using occupation or profession) for both parents, might be an additional strength in the neurodevelopment study. Finally, our study incorporated and assessed a single developmental domain rather than a set of childhood developmental domains, as many previous studies have done. This is very relevant to understanding childhood development in a holistic way and to address it appropriately. 

Major strengths of this study include the use of a comprehensive and validated tool to assess the developmental function of infants and young children. Also, data were collected in detail by the same and highly trained research team. Food processing and diet diversity were evaluated using a two-day 24 h dietary recall, from non-consecutive days, completed by the parents and educators. Although a single 24 h dietary recall questionnaire seems to be trustworthy to capture important information about individual food and drink intake, the two-day 24 h dietary recall is able to account for intake variations and to identify irregularly consumed foods and beverages. In addition, the dietary data might be affected by a recall bias, since this questionnaire implies the report of food and beverages consumed in the prior 24 h, resorting to memory. Nevertheless, the two-day food records were obtained and completed by the parents and educators from the childcare centers, who are able to report the foods and beverages consumed by toddlers in the prior 24 h more easily. Lastly, and to our knowledge, this is the first study performing the evaluation of the association between unprocessed and minimally processed foods, dietary diversity and neurodevelopment in Portuguese toddlers. Also, we targeted a highly sensitive developmental period characterised by multiple vulnerabilities. It is also the first work suggesting a mechanism through which diet, characterised by high energetic contribution to total energy intake of unprocessed and minimally processed foods and high dietary diversity, might interfere with neurodevelopment in the toddler period.

This study implies the role of two important diet dimensions—food processing and dietary diversity—on neurodevelopment in toddlers. As a result, it is critical to conduct prospective and experimental studies to evaluate changes over time, as well as the cumulative effects of dietary habits and diet quality. To better address the effects of the diet, repeated assessments are required, and the diverse dietary sources of unprocessed and minimally processed foods and the food groups that most contributed to total energy intake should also be analysed. Given the recognised importance of the childhood period, as well as the possible implications for proper cognitive development, future research in this field may advocate feasible targets and major directions for early public health prevention, education and intervention through future programs about the rule of dietary intake on neurodevelopment for parents, caregivers, educators and health professionals.

## 5. Conclusions

This study intended to estimate the associations between dietary factors and neurocognitive development in toddlers. Our findings suggest that the higher energy contribution of unprocessed and minimally processed foods and higher dietary diversity are associated with higher neurodevelopment in girls, but not in boys. This should be further studied as a possible early link between dietary factors and neurodevelopment. Promotion of healthy eating, consumption of unprocessed and minimally processed foods and dietary diversity can be promising in improving neurocognitive development and might help to introduce clinical guidelines and public health recommendations for toddlers’ nutrition. 

## Figures and Tables

**Table 1:** Summary of participants’ characteristics by sex (*n* = 212).

	Girls (*n* = 110)	Boys (*n* = 102)	Total (*n* = 212)	*p*-Value
Age (months), mean ± SD	23.61 ± 6.13	23.94 ± 6.56	23.77 ± 6.33	0.704 ^1^
TEI (kcal/day), median (25th–75th)	1030.01 (912.52–1192.41)	1100.34 (975.97–1281.30)	1077.33 (930.94–1236.42)	0.111 ^1^
Breastfeeding ^a^, *n* (%)	19 (17.3)	12 (11.8)	31 (14.6)	0.257 ^2^
Infant formula ^b^, *n* (%)	25 (22.7)	23 (22.5)	48 (22.6)	0.975 ^2^
Sleeping time (hours), mean ± SD	10.31 ± 0.82	10.28 ± 0.63	10.30 ± 0.72	0.793 ^1^
Mother education, *n* (%)				0.607 ^2^
Basic/High School	43 (39.4)	36 (36.0)	79 (37.8)	
University	66 (66.6)	64 (64.0)	130 (62.2)	
Bayley cognitive score, mean ± SD	10.04 ± 2.72	9.56 ± 2.60	9.81 ± 2.67	0.193 ^1^
Bayley’s cognitive categories, *n* (%)				0.448 ^2^
Below median cognitive scaled score (score < 10)	45 (40.9)	47 (46.1)	92 (43.4)	
Above median cognitive scaled score (score ≥ 10)	65 (59.1)	55 (53.9)	120 (56.6)	

^1^ Independent sample *t*-test was used for continuous normally distributed variables; ^2^ X^2^-square test was used for categorical variables; ^a^ toddler was breastfed; ^b^ toddler was fed with infant formula. Notes: SD—Standard Deviation; % percentage; TEI—Total Energy Intake.

**Table 2:** Summary of dietary information by sex.

	Girls (*n* = 110)	Boys (*n* = 102)	Total (*n* = 212)	*p*-Value
NOVA classification (% kcal TEI) Including infant formulas				
NOVA 1, mean ± SD	58.15 ± 13.02	55.95 ± 12.60	57.09 ± 12.83	0.219 ^1^
NOVA 2, mean ± SD	8.20 ± 3.56	7.54 ± 3.16	7.89 ± 3.38	0.163 ^1^
NOVA 3, mean ± SD	13.15 ± 7.69	11.23 ± 7.69	12.22 ± 7.73	0.076 ^1^
NOVA 4, mean ± SD	20.44 ± 13.93	25.29 ± 14.73	22.78 ± 14.49	0.016 ^1^
NOVA classification (% kcal TEI) Excluding infant formulas				
NOVA 1, mean ± SD	61.11 ± 11.91	59.43 ± 11.77	60.31 ± 11.85	0.304 ^1^
NOVA 2, median (25th–75th)	8.62 (5.90–10.89)	8.00 (6.00–10.20)	8.26 (5.94–10.45)	0.321 ^3^
NOVA 3, mean ± SD	13.63 ± 7.96	11.37 ± 7.92	12.54 ± 8.00	0.039 ^1^
NOVA 4, mean ± SD	16.55 ± 12.05	21.15 ± 12.44	18.77 ± 12.42	0.007 ^1^
Dietary diversity score, mean ± SD	4.99 ± 0.79	5.07 ± 0.76	4.91 ± 0.81	0.257 ^2^
Dietary diversity categories, *n* (%)				
Below median score (<5)	79 (37.3)	37 (33.6)	42 (41.2)	
Above median score (≥5)	133 (62.7)	73 (66.4)	60 (58.8)	

^1^ Independent sample *t*-test was used for continuous normally distributed variables; ^2^ X^2^-square test was used for categorical variables; ^3^ Mann–Whitney test was used for non-normally distributed variables. Notes: SD—Standard Deviation; % percentage; TEI—Total Energy Intake.

**Table 3:** Association between energetic contribution to total energy intake of NOVA 1 and toddlers’ neurodevelopment.

NOVA Classification: aOR (95%CI)	NOVA 1 Above Median Cognitive Scaled Score Crude Model	*p*-Value	NOVA 1 Above Median Cognitive Scaled Score Adjusted Model	*p*-Value
Model 1				
All participants	1.04 (1.01; 1.06)	0.004	1.05 (1.01; 1.09)	0.036
Girls	1.04 (1.01; 1.08)	0.015	1.06 (1.01; 1.11)	0.018
Boys	1.03 (0.99; 1.06)	0.134	1.03 (0.99; 1.07)	0.208
Model 2				
All participants	1.02 (1.00; 1.05)	0.034	1.03 (1.00; 1.06)	0.037
Girls	1.04 (1.01; 1.08)	0.008	1.06 (1.01; 1.11)	0.024
Boys	1.00 (0.97; 1.03)	0.956	1.02 (0.98; 1.07)	0.299
Model 3				
All participants	1.04 (1.01; 1.06)	0.004	1.03 (1.00; 1.06)	0.032
Girls	1.04 (1.01; 1.08)	0.015	1.05 (1.00; 1.11)	0.033
Boys	1.03 (0.99; 1.06)	0.134	1.02 (0.98; 1.07)	0.239
Model 4				
All participants	1.02 (1.00; 1.05)	0.034	1.03 (1.00; 1.06)	0.041
Girls	1.04 (1.01; 1.08)	0.008	1.05 (1.00; 1.11)	0.043
Boys	1.00 (0.97; 1.03)	0.956	1.02 (0.98; 1.06)	0.338

Logistic regression models were employed to estimate the associations between Bayley’s cognitive categories and high energetic contribution to total energy intake of NOVA 1. Notes: significant differences were defined with an α-value of less than 5%, 95% confidence interval, (*p* < 0.05); CI—Confidence Interval; %—percentage; aOR—adjusted odds ratio. Model 1: association between NOVA 1, excluding %kcal of infant formulas to total energy intake and Bayley’s cognitive categories. Adjusted model for sex, age, breastfeeding, toddler’s sleeping time and mothers’ education; Model 2: association between NOVA 1, including %kcal of infant formulas to total energy intake and Bayley’s cognitive categories. Model adjusted for sex, age, breastfeeding, percentage energetic contribution to total energy intake of infant formulas, toddler’s sleeping time and mother’s education; Model 3: association between NOVA 1, excluding %kcal of infant formulas to total energy intake and Bayley’s cognitive categories. Adjusted model for sex, age, toddler’s sleeping time and mother’s education; Model not adjusted for breastfeeding. Model 4: association between NOVA 1, including %kcal of infant formulas to total energy intake and Bayley’s cognitive categories. Model adjusted for sex, age, percentual energetic contribution to total energy intake of infant formulas, toddler’s sleeping time and mother’s education; Model not adjusted for breastfeeding.

**Table 4:** Association between Minimum Dietary Diversity and toddlers’ neurodevelopment.

Minimum Dietary Diversity: aOR (95%CI)	Food Diversity Above Median Cognitive Scaled Score Crude Model	*p*-Value	Food Diversity Above Median Cognitive Scaled Score Adjusted Model	*p*-Value
Model 1				
All participants	1.47 (0.84; 2.58)	0.177	1.28 (0.65; 2.49)	0.476
Girls	2.26 (1.01; 5.06)	0.048	3.15 (1.05; 9.48)	0.041
Boys	0.94 (0.43; 2.08)	0.887	0.61 (0.23; 1.63)	0.329
Model 2				
All participants	1.47 (0.84; 2.58)	0.177	1.24 (0.63; 2.43)	0.533
Girls	2.26 (1.01; 5.06)	0.048	3.18 (1.12; 9.08)	0.030
Boys	0.94 (0.43; 2.08)	0.887	0.65 (0.25; 1.70)	0.382

Logistic regression models were employed to estimate the associations between Bayley’s cognitive categories and Minimum Dietary Diversity. Notes: significant differences were defined with an α-value of less than 5%, 95% confidence interval, (*p* < 0.05); CI—Confidence Interval; %—percentage; aOR: adjusted odds ratio. Model 1: association between minimum dietary diversity and Bayley’s cognitive categories. Adjusted model for sex, age, percentual energetic contribution to total energy intake of infant formulas, toddler’s sleeping time and mother’s education; Model also adjusted for breastfeeding. Model 2: association between Minimum Dietary Diversity and Bayley’s cognitive categories. Model adjusted for sex, age, percentual energetic contribution to total energy intake of infant formulas, toddler’s sleeping time and mother’s education; Model not adjusted for breastfeeding.

## Data Availability

Data is available upon request. The data are not publicly available due to confidentiality and privacy considerations.
